# Drug-Related Glomerular Phenotypes: A Global Pharmacovigilance Perspective

**DOI:** 10.3390/jcm13164869

**Published:** 2024-08-18

**Authors:** Alexandre Baptista, Ana M. Macedo, Ana Marreiros, André Coelho, Mark A. Perazella

**Affiliations:** 1 School of Medicine and Biomedical Sciences, Algarve University (UAlg), 8005-139 Faro, Portugal; anamoitademacedo@gmail.com (A.M.M.); ammarreiros@ualg.pt (A.M.); 2 Algarve Biomedical Centre (ABC), 8005-139 Faro, Portugal; 3 Health & Technology Research Center, Escola Superior de Tecnologia da Saúde de Lisboa, Instituto Politécnico de Lisboa, 1990-096 Lisboa, Portugal; andre.coelho@estesl.ipl.pt; 4 School of Medicine, Yale University, New Haven, CT 06520, USA; mark.perazella@yale.edu

**Keywords:** pharmacovigilance, drug therapy, pharmacology, drug-related side effects and adverse reactions, glomerular diseases

## Abstract

**Introduction:** Adverse drug reactions are a significant problem in modern society, stemming from the increase in prescribed medications, over-the-counter drugs, and overall polypharmacy. Glomerular disorders are one of the frequently reported renal conditions associated with medication use. VigiBase is a significant tool for evaluating events associated with drug use, and, to the authors’ knowledge, no study has yet assessed this database to identify the primary medications associated with glomerular disorders. **Materials and Methods:** We collected data from VigiBase for 54 years and evaluated data based on global frequencies, disproportionality (IC_025_ values), nephrotoxic potential, and physiopathological mechanisms. **Results:** Over the evaluation period, 33.932.051 spontaneous notifications of adverse drug reactions reported in VigiBase were assessed, from which 106.775 notifications of drug-associated glomerular disorders were extracted. The isolated medications were classified as ‘potential nephrotoxins’ (47.0%), with 40% of the medications lacking scientific references to report any association with the development of glomerular disorders. Among the evaluated medications, Inotersen (IC_025_ of 8.3), Penicillamine (IC_025_ 6.8), Bevacizumab (IC_025_ 5.9) and Lenvatinib (IC_025_ 5.4) were identified as having the strongest association with these glomerular disorders. For medications classified as ‘non-nephrotoxic’, a high disproportionality index was observed, suggesting drugs that might be considered as new potential nephrotoxins. **Conclusions:** Drug-induced glomerular disorders were significantly associated with medications that had no established nephrotoxic role but demonstrated a high disproportionality index in VigiBase. These newly alleged nephrotoxic drugs warrant further evaluation in dedicated studies to assess their true nephrotoxic potential.

## 1. Introduction

The ability to prescribe medication is a cornerstone of modern medicine, requiring a careful balance between therapeutic benefits and the potential adverse effects arising from drug use [1]. Drug-induced renal diseases are notably prevalent, prompting Mehta et al. to delineate four principal phenotypes of renal manifestations attributable to pharmacological interventions [2].

Among these, various forms of glomerular diseases are increasingly observed as complications of several medications [3]. While the explicit role of drugs in the pathogenesis of glomerular injury is not fully elucidated, their impact is likely substantial [4]. Medications affect various cellular components of the glomerulus, including visceral epithelial cells (podocytes), endothelial cells, and mesangial cells [5]. A variety of pharmaceuticals, such as interferon, bisphosphonates, non-steroidal anti-inflammatory drugs, antiplatelet agents, chemotherapeutic agents, and antiangiogenic drugs, have been implicated in iatrogenic glomerular injury [5].

Pharmacovigilance remains an indispensable tool in the detection, evaluation, understanding, and prevention of adverse drug effects, with active monitoring and reporting of adverse drug reactions being paramount [6].

This study leverages spontaneous notifications from one of the largest global databases of adverse drug reactions to elucidate the drugs most frequently associated with glomerular disorders—VigiBase.

VigiBase was created in 1968 as a result of the thalidomide crisis [7] and serves as a repository of spontaneous notifications regarding adverse drug reactions. It is integrated into the WHO Programme for International Drug Monitoring (WHO PIDM). VigiBase serves its primary users, namely national pharmacovigilance centers and the Uppsala Monitoring Centre, by providing essential information on adverse drug reactions. This role makes VigiBase a vital hub in pharmacovigilance, allowing the generation of new hypotheses regarding drug-related issues and enhancing knowledge about specific characteristics of adverse drug reactions, such as time to onset, progression, associated clinical features, and outcomes. The importance of VigiBase in the core of pharmacovigilance is further highlighted by the fact that it is the largest global source of patient safety data [8].

The data in VigiBase come from the migration of spontaneous adverse reaction reports submitted to each national pharmacovigilance center, which are then migrated to VigiBase. Currently, more than 140 countries contribute to this process. Each notification is evaluated by pharmacists and medical experts who assess the information contained in each report, establishing a potential causal link between the reported manifestation and the suspected drug(s) [9].

We aim to highlight these drugs by their frequency of association, the strength of this association with glomerular disorders, and their recognized nephrotoxic potential, as corroborated by scientific references. This endeavor seeks to contribute foundational data to the broader understanding of drug-associated glomerular disturbances.

## 2. Materials and Methods

We conducted a descriptive analysis of the world’s largest database for adverse drug reaction notifications, VigiBase, following approval from the review board. This comprehensive database aggregates spontaneous reports from a wide array of participating countries, maintaining complete anonymity of data. The dataset spans from 1968 to 2022. Rigorous measures were employed to exclude any duplicate notifications, with each report assigned a unique identification number for precise referencing. The notifications offer extensive details, including anonymized patient information, notifier details, the severity of the adverse reaction, the implicated medication, and a detailed description of the reaction.

Data collection involved filtering notifications using appropriate MedDRA terms (see Supplementary Materials). Each medication was identified by its active ingredient according to WHODrug nomenclature standards and categorized into pharmacological classes based on the WHO’s Anatomical Therapeutic Chemical (ATC) classification system. This facilitated a systematic analysis in relation to specific pharmacological categories.

For this study, we employed the World Health Organization’s IC_025_ index to identify medications with a disproportionate number of notifications. The IC compares the observed frequency of a specific adverse reaction associated with a medication against its expected frequency in the general population. A positive IC suggests that the adverse reaction is reported more frequently than expected, indicating a potential association. By automatically adjusting for expected frequencies, the IC minimizes random variations and reduces the likelihood of false positives caused by statistical fluctuations, thus only emphasizing statistically significant associations. The IC filters out irrelevant data, distinguishing and disregarding chance associations to reduce false positives. The IC_025_, representing the lower limit of the 95% confidence interval for the IC, provides a measure of certainty that an observed medication-adverse reaction association is not occurring by chance. The IC_025_ is already integrated into VigiBase, and the data was extracted directly from it.

Also, each drug was evaluated in 5 different bibliographic sources regarding its role in the formation of kidney stones (two databases [10,11], one website [12], and two reference books [13,14]). A bibliographic score (BS) was developed and considered a surrogate for each drug’s lithogenic role (0—not lithogenic; 1–2—potentially lithogenic; and 3–5—lithogenic). For each medication, we counted the number of references mentioning the phenotype as an adverse effect of the drug. The score assigned to each medication corresponded to the total number of sources referencing the adverse event. For example, if a medication had 2 sources mentioning the adverse event, then the BS for that medication would be 2.

With this score in mind, the main medications involved and reported in the obtained notifications were evaluated regarding the pathophysiology of their nephrotoxicity, whenever available.

## 3. Results

Between 1968 and 2022, VigiBase recorded a total of 33,932,051 spontaneous notifications of adverse drug reactions. Of these, 106,775 notifications pertained to drug-associated glomerular disorders, constituting 0.32% of all reports during the analyzed period. These notifications predominantly involved male consumers (52.9% for males; 42.2% for females; 4.9% of notifications did not report the sex) and individuals aged 45–64 years (22.8%), with this sample presenting a mean age of 48.5 ± 7.42 years. The USA was the main reporter (47.5%), followed by Italy (6.0%) and Germany (5.5%), and the notifications were reported by physicians (36.5%), consumers (23.5%), and other healthcare professionals (11.3%).

Hematuria emerged as the most frequently reported glomerular disturbances, documented in 52.5% of the cases, followed by proteinuria reported in 13.3%, with this presenting as nephrotic syndrome in 6.5% of the notifications. Pyrexia was the most common accompanying complaint, present in 6.3% of the notifications, followed by fatigue (5.8%) and nausea (4.7%) (see Table 1).

Among the main drugs assessed, the number of drugs classified as potential new nephrotoxins (BS 0) was very similar to those classified as potential nephrotoxins (BS 1–2) (39.1% for new nephrotoxins and 47.8% for potential nephrotoxins). In fact, only 13.1% of the main drugs evaluated were considered known nephrotoxins based on the bibliographic score (BS 3–5) (see Table 2).

From an absolute perspective, Rivaroxaban, the COVID-19 vaccine (77.6% Tozinameran; 14.6% Elasomeran; 5.4% ChAdOx1 nCoV-19; 2.4% others), Warfarin, and Acetylsalicylic Acid were described in 7.5%, 7.1%, 5.4%, and 5.2% of the notifications, respectively (see Table S2 of Supplementary Materials). This distribution explains why the ATC class B was the most frequently reported (29.6%), followed by ATC class L (25.1%), and ATC class J (20.9%) (see Table S1 of Supplementary Materials).

Apart from the COVID-19 vaccine, which only had an IC_025_ of 1.9 for proteinuria, the others showed values indicative of a significant association with glomerular disorders, as seen with Rivaroxaban (IC_025_ of 4.5 for Hematuria), Acetylsalicylic Acid (IC_025_ of 3.9 for Hematuria), and Warfarin (IC_025_ of 4.6 for Hematuria). Besides these, we observed the presence of other drugs which, although not as frequently reported, show a significant association with glomerular disorders, such as Inotersen (IC_025_ of 8.3 for increased urine protein/creatinine ratio), Penicillamine (IC_025_ of 6.8 for nephrotic syndrome), Bevacizumab (IC_025_ of 5.9 for proteinuria), and Lenvatinib (IC_025_ of 5.4 for proteinuria), among others (see Table S3 of Supplementary Materials).

Among the main drugs evaluated, we identified Lansoprazole, Heparin, Acetylsalicylic Acid, and Tenofovir as lacking bibliographic references according to our score, suggesting they may be potential new nephrotoxins related to glomerular disorders.

For the medications evaluated, an increasing average IC_025_ was observed as we transitioned from ‘non-nephrotoxic’ (average IC_025_ of 3.03) to ‘potentially nephrotoxic’ (average IC_025_ of 3.66) and then to those categorized as nephrotoxic (average IC_025_ of 3.87).

## 4. Discussion

Our study evaluated VigiBase, one of the largest databases of spontaneous notifications of adverse drug reactions, over a period of 54 years. The aim was to identify, qualify, and compare the principal medications associated with the development of glomerular disorders with bibliographic references, supplemented by the pathophysiological principles considered for such an association.

To the best of the authors’ knowledge, this is one of the first studies to utilize such a database for evaluating drug-associated glomerular disturbances. The small proportion of isolated notifications (0.32%) reflects the challenges in identifying this type of renal alteration or in associating them with drug use.

To achieve the outlined objectives, the authors conducted a descriptive evaluation of filtered data from VigiBase, and utilized an integrated disproportionality index (IC_025_), which enabled the assessment of the ‘strength’ of the association between drug and disorder. Additionally, a bibliographic score was developed to determine the documented nephrotoxic potential of each principal medication. This approach not only facilitated the identification of already known nephrotoxins but also underscored the nephrotoxic roles of certain drugs (high IC_025_; high BS) and pinpointed potential new nephrotoxins (high IC_025_; low BS).

The data obtained indicate that individuals aged between 45–64 years were the most frequently reported in notifications of drug-induced glomerular diseases, with the male sex also being predominant. This is in line with data obtained from the literature [15,16].

In relation to renal manifestations associated with medication use and expressed in VigiBase spontaneous notifications, ‘PT Hematuria’ or ‘Presence of blood in urine’ were the most frequently reported, with respective frequencies of 52.5% and 17.4% in the notifications obtained. This finding must be interpreted cautiously, as the clinical manifestation of hematuria may not necessarily reflect an adverse drug reaction (ADR), but rather a side effect of the medication. Indeed, the presence of 7 anticoagulants and/or antiplatelet agents within the top 10 could contribute to this manifestation (non-glomerular diseases-related hematuria). Literature reports an incidence of hematuria associated with anticoagulant use at 26.7%, decreasing to 1.1% in thrombotic event prophylaxis, with antiplatelet agents being 76 times less likely to cause hematuria compared to anticoagulants [17].

However, among the top 10 drugs dominated by anticoagulants (refer to Table S2 in the Supplementary Materials), three medications outside the ATC B class are noteworthy: the COVID-19 vaccine (7.1%), Bevacizumab (2.9%), and Adalimumab (1.3%). These drugs are associated with the development of proteinuria, thrombotic microangiopathy, or IgA nephropathy.

We demonstrated that the main drugs evaluated exhibited a significantly elevated average IC_025_ of 3.44, suggesting already a strong preferential association with glomerular disorders compared to other adverse reactions.

Examining the ATC classes, the three most involved ATC drug classes—ATC classes B, L, and J—showed average IC025 values of 4.2, 3.0, and 1.87, respectively, illustrating a stronger linkage to glomerular disorders in classes B and L, and a somehow lower association with class J drugs.

Among the principal medications identified, Inotersen (IC_025_ of 8.3), Penicillamine (IC_025_ of 6.8), Bevacizumab (IC_025_ of 5.9), and Lenvatinib (IC_025_ of 5.4) stand out, all of which are strongly associated with the development of proteinuria.

Notably, the association between drugs and glomerular disturbances is distinctly evident in 39.1% of the drugs that exhibit a high association with these disorders (IC_025_ 3.03) but are not supported by any of the evaluated bibliographic sources (BS = 0), potentially identifying these drugs as new nephrotoxins.

Among these top medications whose bibliographic scores were evaluated and are potential new nephrotoxins, we highlight Lansoprazole (IC_025_ 4.4), Omeprazole (IC_025_ 3.1), Tenofovir (IC_025_ 2.8), and Adalimumab (IC_025_ 1.9). Lansoprazole has been associated with the development of interstitial nephritis [18] and, consequently, acute kidney injury [19], but the literature does not directly associate it with the development of proteinuria. In this case, proteinuria may be associated, in the initial phase, with tubular proteinuria associated with the development of interstitial nephritis [20,21]. The mechanism associated with the development of interstitial nephritis and, thus, proteinuria from Lansoprazole, like other drugs, involves drug binding to the tubular basement membrane (TBM), acting as a hapten or mimicking an antigen normally present in the TBM or interstitium, inducing an immune response [22]. Regarding Tenofovir, although our score suggested it as a potential new nephrotoxin, further targeted investigation revealed that proteinuria has been observed in 27% of patients treated with Tenofovir [23], with each year of use associated with a 34% increased risk of developing proteinuria [24,25]. This proteinuria, again tubular in origin, results from direct toxicity of Tenofovir at this level, mainly in the proximal tubule, although the mechanism is not fully elucidated, it may result from mitochondrial dysfunction [26]. Finally, among medications with a BS of 0 and an increasing association with proteinuria, we also highlight Adalimumab, a drug, in our sample, linked to the development of IgA Nephropathy [27,28]. In fact, this association still seems weak, both because the IC_025_ is only 1.9 and because the few references found are related to isolated clinical cases.

Changing to the hematuria phenotype and focusing now on medications with a BS of 0–all from the ATC B class, we highlight acetylsalicylic acid with an IC_025_ of 3.9. This medication, despite being a platelet function inhibitor, is not associated with an increase in microscopic hematuria depending on its use [29], so hematuria associated with aspirin should result from different mechanisms. These conclusions are contradicted by other authors who demonstrate a significant increase in complications associated with hematuria [30]. This association has also been described in population studies, with aspirin being associated with the development of hematuria in 24.54% of elderly consumers [31]. Indeed, in a case series evaluation, urothelial disorders were one of the main causes associated with aspirin use, namely tumor conditions or hemorrhagic cystitis [32], and, therefore, hematuria concurrent with an antiplatelet agent should always prompt an appropriate investigation for hematuria. Enoxaparin, a well-known anticoagulant, despite the high predicted association with hematuria (IC_025_ 4.2), sees this association as poorly described, even with a more in-depth analysis. In fact, the American Urological Association, in its guidelines on hematuria, considers the evidence strength for this statement as Grade C [33].

Focusing on medications with a higher degree of association with glomerular disorders, we highlight Inotersen (IC_025_ of 8.3) for an increase in the protein/creatinine ratio, one of the highest in the entire VigiBase. For this drug, the BS was 2, suggesting its potential nephrotoxicity, with literature references indicating reversible development of proteinuria [34], and associations with segmental and focal glomerulosclerosis (FSGS) [35], although FSGS has been showed in only 3% of patients receiving this drug [34]. Proteinuria associated with antisense oligonucleotides may also result from their tubular accumulation affecting protein tubular reabsorption without any associated tubular injury [34].

Also, among drugs with ‘potential nephrotoxicity’, Penicillamine shows a strong association in this drug-reaction pair, with an IC_025_ of 6.8 for nephrotic syndrome. Its summary product characteristics (SPC) indicate that its association with the emergence of nephrotic syndrome is very common (>1 in 10 patients) [36], particularly in female patients and generally after 7 months of therapy [37], occurring in approximately 5–30% of patients [38]. Indeed, the mechanisms by which Penicillamine associates with the development of proteinuria result from induction by this drug of membranous glomerulonephritis [39,40].

Furthermore, Rivaroxaban (IC_025_ 4.5 for hematuria), Acenocoumarol (IC_025_ 4.8 for hematuria), Dabigatran (IC_025_ 4.0 for hematuria), and Warfarin (IC_025_ 4.6 for hematuria) all demonstrated significant associations with the aforementioned glomerular disorders and received a bibliographic score (BS) of 2, classifying them as ‘potential nephrotoxins’.

Bevacizumab was identified as the drug with the highest literary evidence of glomerular nephrotoxicity, exhibiting a BS = 3 and an IC_025_ of 5.9 for proteinuria and 3.9 for nephrotic syndrome, reflecting its strong connection to this phenotype. Literature references link it to glomerular microangiopathy [41] and the development of proteinuria [42] or nephrotic syndrome [43], with thrombotic microangiopathy being the characteristic histological pattern [44], although a few cases of IgA vasculitis with nephritis have been reported [45]. Lenvatinib, already recognized as nephrotoxic (BS = 3), also shows a strong connection to this glomerular disorder with an IC_025_ of 5.4 for proteinuria, a condition noted in its SPC [46] and other references [47]. The mechanisms appear to be similar to those of post-exercise proteinuria, with disruption of the podocyte-endothelial VEGF signaling axis, subacute thrombotic microangiopathy, and podocyte protein junction downregulation [48].

The future of research on adverse drug reactions may lie in pharmacovigilance studies integrated with electronic medical records, enabling retrospective analysis of identified adverse drug reactions [49]. However, prior genetic determination before the administration of drugs could also provide more information and safety in the realization of certain medications [50]. Additionally, artificial intelligence and machine learning will aid in the early identification of these reactions [51], allowing for a significant reduction in toxicity [52].

This study has several strengths. By obtaining its data from an already structured and anonymized database, based on the MedDRA and WHOdrug dictionaries, it significantly reduced biases resulting from the need to be processed by the authors. Its global origin strengthens the results obtained and reduces biases from local clinical practices. The scope of the data allows the results to be considered as potential signals in the identification or reinforcement of possible nephrotoxic medications, enhancing their recognition.

However, this study also has limitations. As it results from data derived from spontaneous notifications, the data exhibit biases of notification, which may reflect rare or atypical manifestations. As an analysis of spontaneous notifications, it is also not possible to establish a causal link between the reported medication and the identified manifestation. Representing the expression of spontaneous notifications, the vast majority with multiple identified manifestations and several drugs involved, it is not possible to assume that either the manifestation or the drug represents the true adverse reaction or the iatrogenic medication. In this sense, some medications may not be inherently nephrotoxic, but the phenotype attributed to them may result from all the aforementioned biases. We should also consider the bibliographic score as a study limitation. The developed bibliographic score, despite attempts to be comprehensive, is limited to the search of only 5 bibliographic sources, which may result in missing some references regarding the adverse reaction in question. This type of study also presents significant biases. These notifications can originate from trained healthcare professionals as well as from consumers or non-medical individuals like family or friends, thus reducing the accuracy of the correct medication–phenotype pair. Underreporting biases are also a concern, as they can lead to an underestimation of the frequency of adverse drug reactions. This may result in medications being falsely portrayed as safer than they actually are or affecting disproportionality indexes. Selective reporting bias is another issue, where newer (Weber effect) or severe adverse reactions, or those linked to well-known medications, are more likely to be reported, creating a skewed perception of risk. Moreover, due to the frequent involvement of multiple medications in the reports collected, determining the true “suspected drug” can be challenging and may lead to misattribution. Lastly, recall and information biases can distort clinical information provided in reports, potentially attributing causality incorrectly to a different medication.

Despite these potential limitations, this study sheds light on the medications most frequently associated with various glomerular disorders, highlighting numerous drugs both for their strong associative links to these glomerular alterations and for identifying potential new nephrotoxic agents. With this study, the authors aim to put into perspective the main medications reported and associated with various types of glomerular disease manifestations, drawing clinicians’ attention to the increased vigilance needed for patients receiving such medications. This, in turn, can help enhance medication safety.

## 5. Conclusions

Glomerular disease, while infrequently reported globally, stands out among various renal disorders in VigiBase. A significant percentage of the identified and categorized medications were classified as ‘non-nephrotoxic’ according to our classification system, yet they are notable for their strong association with this type of glomerular disorders. A wide range of pathophysiological mechanisms justifies the association between these evaluated medications and the phenotype in question.

However, these medications require further evaluation to ascertain the true risk of developing glomerular disorders among these potential new nephrotoxins.

## Figures and Tables

**Table 1:** Main MedDRA terms reported and co-reported in the ‘Glomerular Disorders’ phenotype associated with drug use.

Reported Preferred Terms (MedDRA)		Co-Reported Preferred Terms (MedDRA)	
Hematuria	52.5%	Fever	6.3%
Blood urine present	17.4%	Fatigue	5.8%
Proteinuria	13.3%	Nausea	4.7%
Nephrotic syndrome	6.5%	Urinary tract infection	4.4%
Protein urine present	3.8%	Headache	4.2%
Albuminuria	3.1%	Diarrhea	4.2%
White blood cells urine positive	2.3%	Acute kidney injury	4.0%
Culture urine negative	1.5%	Vomits	3.8%
Blood urine	1.4%	Anemia	3.8%
Protein urine	1.3%	Pain	3.8%

**Table 2:** Drugs most associated with glomerular disturbances or having a higher disproportionality index and their relationship with the bibliographic score.

Active Ingredient	% Notifications	ATC Class	IC_025_	Phenotype	BS
Bevacizumab	2.9%	L	5.9	Proteinuria	3
Ibuprofen	0.8%	M	0.3	Proteinuria	3
Lenvatinib	0.4%	L	5.4	Proteinuria	3
Apixaban	2.4%	B	3.5	Renal Hemorrhage	2
Clopidogrel	2.1%	B	4.0	Hematuria	2
Inotersen	0.3%	N	8.3	Proteinuria	2
Penicillamine	0.5%	M	6.8	Nephrotic Syndrome	2
Acenocoumarol	0.8%	B	4.9	Hematuria	1
Dabigatran	2.5%	B	4.0	Hematuria	1
Lithium	0.1%	N	2.8	Nephrotic Syndrome	1
Esomeprazol	0.7%	A	0.9	Hematuria	1
Selumetinib	0.1%	L	3.4	Proteinuria	1
Rivaroxaban	7.5%	B	4.5	Hematuria	1
Warfarin	5.4%	B	4.7	Hematuria	1
Acetylsalicylic Acid	5.2%	B	3.9	Hematuria	0
Adalimumab	1.3%	L	1.9	IgA Nephropathy	0
COVID-19 vaccine	7.1%	J	1.9	Proteinuria	0
Enoxaparin	1.7%	B	4.2	Hematuria	0
Heparin	1.0%	B	4.2	Hematuria	0
Lansoprazole	1.0%	A	4.4	Proteinuria	0
Omeprazole	1.0%	A	3.1	Proteinuria	0
Pneumococcal vaccine	1.0%	J	0.9	Urinary sediment abnormal	0
Tenofovir	0.5%	J	2.8	Proteinuria	0

ATC: Anatomical Therapeutics Class; ATC:A—Alimentary Tract and Metabolism; ATC:B—Blood and Blood forming organs; ATC:J—Antiinfectives of Systemic Use; ATC:L—Antineoplastic and Immunomodulating Agents; ATC:M—Musculo-skeletal system; ATC: N—Nervous System.

## Data Availability

The data will be available for consultation if deemed necessary. The data presented in this study were sourced from VigiBase. The interpretation and reporting of these findings are solely the responsibility of the author(s) and should not be construed as an official policy or interpretation of the Uppsala Monitoring Centre.

## Supplementary Materials

The following supporting information can be downloaded at: https://www.mdpi.com/article/10.3390/jcm13164869/s1, Table S1: Most reported ATC classes in spontaneous notifications of drug-associated glomerular disease–WHODrug classification; Table S2: Most reported active ingredients (WHODrug classification) in spontaneous notifications of drug-associated glomerular disease (top-10); Table S3: Active ingredients (WHODrugs) with the highest disproportionality in drug-associated glomerular disease (top-10).
