# Supplementary material for: Drug-Related Glomerular Phenotypes: A Global Pharmacovigilance Perspective
Source: J Clin Med. 2024 Aug 18;13(16):4869. doi: 10.3390/jcm13164869 (PMC11355908; doi:10.3390/jcm13164869)
Supplement: Supplementary file 1 [file jcm-13-04869-s001.zip › jcm-3152083-supplementary.pdf]

Supplementary Tables

Table S1 – Most reported ATC classes in spontaneous notifications of drug-associated glomerular disease – WHODrug classification.

ATC: B Blood and Blood Forming Organs	29.6%
ATC: L Antineoplastic and Immunomodulating Agents	25.1%
ATC: J Anti-infectives for Systemic Use	20.9%
ATC: A Alimentary Tract and Metabolism	14.4%
ATC: M Musculoskeletal System	12.4%
ATC: S Sensory Organs	12.1%
ATC: N Nervous System	11.8%
ATC: C Cardiovascular System	10.6%
ATC: D Dermatologicals	8.5%
ATC: G Genitourinary System and Sex Hormones	6.6%
ATC: R Respiratory System	4.3%
ATC: H Systemic Hormonal Preparations, Excluding Sex Hormones and Insulins	2.9%
ATC: V Various	2.7%
ATC: P Antiparasitic Products, Insecticides and Repellents	0.8%

Table S2 – Most reported active ingredients (WHODrug classification) in spontaneous notifications of drug-associated glomerular disease (top-10).

AI: Rivaroxaban	7.5%
AI: Covid-19 vaccine	7.1%
AI: Warfarin	5.4%
AI: Acetylsalicylic acid	5.2%

AI: Bevacizumab	2.9%
AI: Dabigatran	2.5%
AI: Apixaban	2.4%
AI: Clopidogrel	2.1%
AI: Enoxaparin	1.7%
AI: Adalimumab	1.3%

Table S3 – Active ingredients (WHODrugs) with the highest disproportionality in drug-associated glomerular disease (top-10)

**Active ingredients**	**$N_{observed}$**	**$N_{expected}$**	**$IC_{0.25}$**
Inotersen	187	0	8.3
Penicilamina	167	1	6.8
Bevacizumab	2258	37	5.9
Lenvatinib	391	8	5.4
Acenocoumarol	857	27	4.9
Tenofovir disoproxil	409	13	4.8
Emtricitabina; Tenofovir disoproxil	508	18	4.7
Varfarina	5436	213	4.6
Edoxaban	702	27	4.6
Rivaroxaban	6547	276	4.5

Legend: $N_{observed}$: number of collected spontaneous notifications in the time period considered; $N_{expected}$: number of expected spontaneous notifications in the time period considered; IC_{025}: World Health Organization disproportionality index.
